# Tracing 2500 years of human betaherpesvirus 6A and 6B diversity through ancient DNA

**DOI:** 10.1126/sciadv.adx5460

**Published:** 2026-01-02

**Authors:** Meriam Guellil, Lucy van Dorp, Lehti Saag, Owyn Beneker, Biancamaria Bonucci, Stefania Sasso, Tina Saupe, Anu Solnik, Helja Kabral, Raili Allmäe, Jessica Bates, Jenna M. Dittmar, Xiangyu Jack Ge, Sarah Inskip, Tõnno Jonuks, Victor N. Karmanov, Valeri I. Khartanovich, Maarten H. D. Larmuseau, Serena Aneli, Craig Cessford, Aivar Kriiska, Marika Mägi, Martin Malve, Natasja De Winter, Mait Metspalu, Luca Pagani, John E. Robb, Toomas Kivisild, Charlotte J. Houldcroft, Christiana L. Scheib, Kristiina Tambets

**Affiliations:** ^1^ Department for Evolutionary Anthropology, University of Vienna, Vienna, Austria.; ^2^ Estonian Biocentre, Institute of Genomics, University of Tartu, Tartu, Estonia.; ^3^ Human Evolution and Archaeological Sciences (HEAS), University of Vienna, Vienna, Austria.; ^4^ UCL Genetics Institute, Department of Genetics Evolution and Environment, University College London, London, UK.; ^5^ Department of Human Genetics, KU Leuven, Leuven, Belgium.; ^6^ Evolutionary Biology Centre, Uppsala University, Uppsala, Sweden.; ^7^ Core Facility, Institute of Genomics, University of Tartu, Tartu, Estonia.; ^8^ Independent Researcher, Kooli 55, Rae vald, Harjumaa 75321, Estonia.; ^9^ Department of Archaeology, University of York, York, UK.; ^10^ Biomedical Affairs, Edward Via College of Osteopathic Medicine, Monroe, LA, USA.; ^11^ McDonald Institute for Archaeological Research, University of Cambridge, Downing Street, Cambridge, UK.; ^12^ Open Targets Genetics, Wellcome Genome Campus, Wellcome Sanger Institute, Hinxton, UK.; ^13^ School of Archaeology and Ancient History, University of Leicester, Leicester, UK.; ^14^ Estonian Literary Museum, Tartu, Estonia.; ^15^ Institute of Language, Literature and History, Komi Science Centre of Ural Branch of Russian Academy of Sciences, Syktyvkar, Russia.; ^16^ Peter the Great Museum of Anthropology and Ethnography (Kunstkamera) of Russian Academy of Sciences, St. Petersburg, Russia.; ^17^ Department of Public Health Sciences and Pediatrics, University of Turin, Turin, Italy.; ^18^ Cambridge Archaeological Unit, University of Cambridge, Cambridge, UK.; ^19^ Institute of History and Archaeology, University of Tartu, Tartu, Estonia.; ^20^ Foundation Osiliana, Tallinn University, Tallinn, Estonia.; ^21^ Archaeological Bureau, Bilzen, Belgium.; ^22^ Department of Biology, University of Padova, Padova, Italy.; ^23^ Department of Archaeology, University of Cambridge, Cambridge, UK.; ^24^ Department of Genetics, University of Cambridge, Cambridge, UK.; ^25^ Department of Zoology, University of Cambridge, Cambridge, UK.; ^26^ St. John’s College, University of Cambridge, Cambridge, UK.

## Abstract

Human betaherpesviruses 6A and 6B (HHV-6A/6B) are DNA viruses, which integrate into the human genome, and are best known to cause “sixth disease.” Despite their recent discovery (1980s), they were speculated to have a much longer history within the human population than modern data suggest. We present the first 11 ancient genomes of HHV-6A and HHV-6B, dating as far back as the 8th to 6th century BCE. We demonstrate that large fractions of current HHV-6 diversity were already established by the 14th century CE. Our data corroborate that HHV-6A/6B integrations stem from ancient founder events. In addition, we show that all known inherited chromosomally integrated HHV-6A clades were already represented in historical populations, confirming that HHV-6A no longer integrates into the germ line within populations of European ancestry and likely endogenized in early human history.

## Teaser

First ancient HHV-6 genomes reveal 2500 years of phylogenetic continuity and provide insights into the endogenization of HHV-6A.

## INTRODUCTION

Human betaherpesviruses 6A (HHV-6A) and 6B (HHV-6B) are two distinct but closely related large double-stranded DNA (dsDNA) viruses restricted to humans. Both viruses were first found in the 1980s from patients with lymphoproliferative disorders (*1*, *2*) and were initially classified as a single species (*3*). Hence, these viruses are relatively understudied, with often unclear effects on human health and limited knowledge of their long-term coevolution with the human host or the age of human association. Today, HHV-6B infects around 90% of all children by the age of two and is best known as the main causative pathogen behind roseola infantum, also known as “sixth disease,” the leading cause of febrile seizures in children, although primary infections have also been reported in adults (*4*). Both HHV-6 viruses can cause mild or asymptomatic primary infections but are also closely associated with disease in immunocompromised individuals with complex disease courses. Whether the link of these conditions with HHV-6 is causal or merely an association remains to be determined. This uncertainty is likely due in part to the relatively recent discovery of the viruses and their ubiquity and lifelong latency within human populations, making it challenging to establish clear causal links. Hence, the viruses have been linked with a wide range of conditions such as transplantation-associated encephalitis (*5*, *6*), graft-versus-host disease (*7*), female infertility (*8*, *9*), myocarditis (*10*, *11*), multiple sclerosis (*12*, *13*), and preeclampsia (*14*, *15*) to name a few.

HHV-6A infections are epidemiologically and immunologically distinct from HHV-6B infections. However, the virus’s involvement in causing disease and its transmission modalities are less well understood. This is because, while HHV-6A is estimated to be phylogenetically older than HHV-6B, it is less prevalent in human populations today (*4*, *16*) and infections occur mostly in adulthood. A factor further complicating the study of the clinical impact of HHV-6A is that due to the high prevalence of acquired HHV-6B infections, most individuals carrying HHV-6A would have been infected by HHV-6B at some point in their life (*17*).

Much like most herpesviruses, the HHV-6 viruses will remain latent in the infected host for life once acquired. However, contrary to all other known human herpesviruses, HHV-6 viruses integrate during the establishment of latency. This is facilitated by the presence of telomere-like terminal repeats in HHV-6AB. Latency can take place in a wide range of tissues, and the viruses can be detected in saliva even after a primary infection following reactivation, which is the main mechanism of spread for roseola infantum (*4*). This usually happens in somatic cells, following an acquired exogenous infection, but rare integration events into germline cells have led to lineages of inherited carriage of HHV-6 sequences within every nucleated cell of the human body, which is estimated to be present in ~0.4 to 1% of the population worldwide (*4*, *18*, *19*). Individuals carrying inherited chromosomally integrated (ici)HHV-6 can then pass on iciHHV-6 vertically to ~50% of their offspring in a Mendelian fashion. Integration sites typically occur within the telomeres (*19*, *20*) and have been shown to affect the length and stability of telomeres in somatic integrations, with some hypothesizing that shorter telomeres might be more frequent integration loci for HHV-6 (*4*, *19*, *21*). The consequences of the viral integration into telomeres are not well understood. However, the location of the integration could have a differential impact on the health of the host (*17*).

This wide range of latency mechanisms has led to markedly different evolutionary dynamics across clades within the virus’s phylogeny, some from acquired infections (acq) (circular/linear genomes, not integrated), noninherited chromosomal integrations (ci) (linear genome, somatic) and inherited chromosomal integrations (ici) (linear genome, germline) (*17*). While acquired infections evolve similarly to nonintegrating infecting viruses (engage in recombination with typically higher mutation rates), integrated versions of the virus show lower genetic diversity in clades, probably due to the higher fidelity achieved by human genomic replication. In addition, integrated versions of the virus are capable of (re)activation, leading to the release of virions (circular genome) (*17*, *22*).

While the seroprevalence of HHV-6B is estimated to be more than 90% in modern populations, this number is hard to verify in ancient and historical populations because of the fact that the most common form of latent carriage would be limited to a small number of somatic cell integrations as opposed to ubiquitous germline-inherited integrations. This makes detection of acq/ciHHV-6B in hard tissue, which is the most common form of tissue available for ancient DNA (aDNA) research, likely difficult. On the other hand, germline chromosomal integrations are ideal for aDNA detection, particularly in samples with high amounts of host DNA. However, inherited infections are much rarer and only present in a small percentage of the human population today (*4*). These carriage rates could have been much lower in the human population at the time of the initial integration rates. It has been speculated that these integrations originate from a small number of human ancestors (*23*). However, modern integrations have also been described for HHV-6B, and no direct data are available to verify the age of the integrated clades described in the literature (*16*). Furthermore, it remains an open question whether HHV-6A is still capable of integrating into the human germ line as previous research has hypothesized that this might no longer be the case (*16*). The timing of such ancient integration events is still unclear, but Aswad *et al.* (*16*) estimated that they occurred more than 50,000 years ago, which would place it around or before the time of the major human migration out of Africa.

Human herpesviruses have been reported in ancient samples [Herpes simplex virus 1 (*24*) and Epstein-Barr virus (*25*)], but full genomes have only been made available for Herpes simplex virus 1, where the addition of dated aDNA genomes enabled a more accurate estimation of the emergence dates of the viral pathogen. This is one of the undisputable advantages of aDNA for the study of dsDNA/single-stranded DNA viruses, which, comparatively to RNA viruses, evolve at much slower rate and therefore can be challenging to study in the context of long-term evolutionary dynamics using solely modern data. In addition, integrated viral sequences remain largely unstudied in the historical population despite the unique opportunity given by aDNA datasets to study the early evolution of endogenous viruses and potentially the early phase of their endogenization in the human germ line. However, the recovery of pathogenic viral DNA from paleogenomic datasets remains rare, recovery rates are affected by a multitude of factors not limited to preservation (*26*), targeted sampling is rarely possible, and full genomes are difficult to recover. The only herpesvirus, which is known to integrate into the host genome, that has been recovered from aDNA to date is the Gallid alphaherpesvirus 2, which causes Marek’s disease in chicken (*27*). Here, we describe the first 11 ancient genomes of two integrating viral pathogens, HHV-6A and HHV-6B, and trace 2500 years of the viruses’ evolution in the European population.

## RESULTS

### Identification and authentication of HHV-6A and 6B

**Fig. 1.**
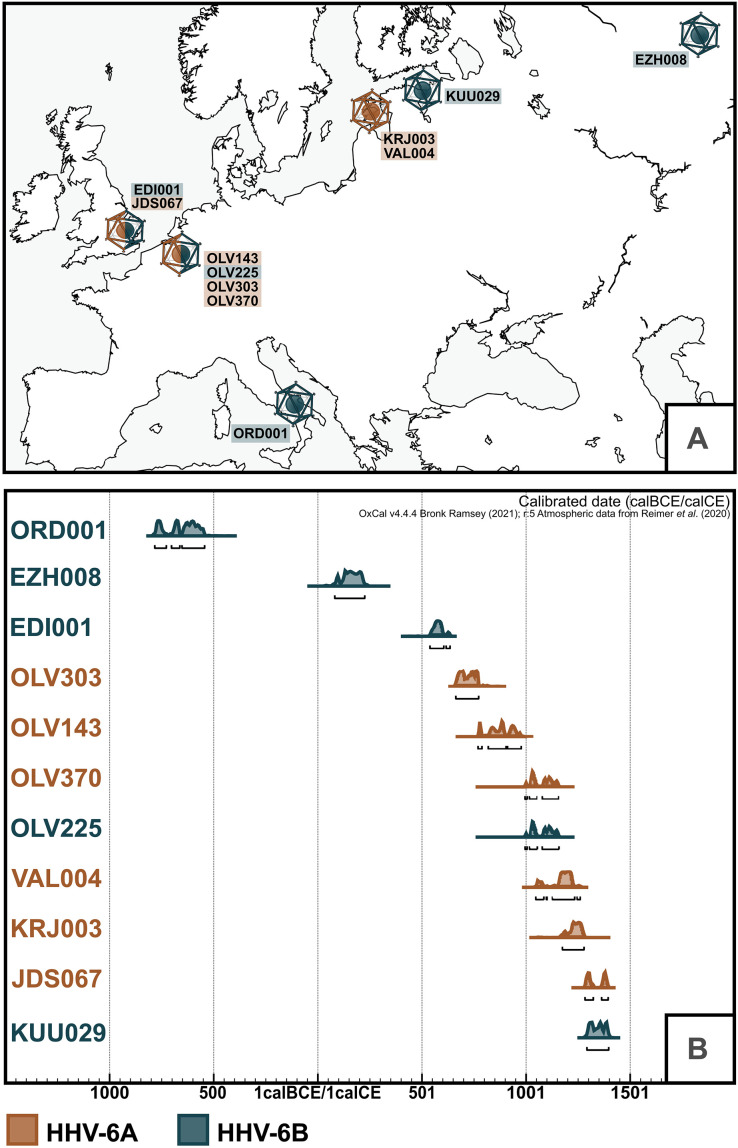
Temporal and geographical range of the sample set. (**A**) Map of Europe depicting all sampling locations and HHV-6 species found at the site. (**B**) OxCal (*75*) plot with calibrated radiocarbon dates for all samples presented in this study. Dates are shown in cal BCE/cal CE (EZH008 dating is likely affected by the freshwater reservoir effect; see Supplementary Text).

During the metagenomic screening of shotgun datasets from larger aDNA population genetics projects involving ~4000 archaeological samples (see table S1), we recovered sequences matching HHV-6 species from a total of 11 human samples spanning from the 8th to 6th century BCE to the 14th century CE (see Fig. 1). Of these, six samples were identified as carrying HHV-6A and five as carrying HHV-6B, through taxonomic classification and comparative mappings (see table S2). Following target enrichment of HHV-6–positive libraries (see Materials and Methods), differentiation from the closely related HHV-7 was further validated by the presence of the *U83* and *U94* genes, which are specific to HHV-6 species and suspected to be involved in the establishment of latency (*4*). Deamination signatures and fragment length (see table S3 and figs. S7 to S9 and S19) were consistent with aDNA.

### Wide temporal, geographic, and cultural range of identified individuals carrying the virus

The samples analyzed in this study stem from a diverse group of individuals in terms of age at death, genetic sex, and geographic provenance (see Table 1). Sample ORD001 stems from a female individual from Ordona (Iron Age Italy), EDI001 from a male aged 16 to 18 years from the Anglo-Saxon site of Edix Hill (England), and JDS067 from a male child excavated at the Hospital of St. John the Evangelist in Cambridge (England). Four of our samples, three female adults and one male adult, originated from a medieval parish graveyard in Sint-Truiden (Belgium). An adult male, an adult woman, and an infant girl were recovered from medieval Estonian sites of Karja, Valjala, and Kukruse, and finally, an older male originated from a site in Ezhol (Komi Republic, Russia). This diversity is further reflected in their cultural and temporal spectrum (see Fig. 1 and Supplementary Text for additional archaeological and osteological data, as well as table S4 for expanded radiocarbon dating data), with dates ranging between the 8th to 6th century BCE and the 14th century CE. While all other HHV-6–positive individuals belonged to farming societies, the individual from Ezhol demonstrates that HHV-6B was also transmitted among taiga hunter-fisher-gatherers. The only site for which more than one individual carrying the viruses could be detected is Sint-Truiden, but only two individuals (OLV225 and OLV370) can be considered contemporaneous.

**Table 1.** Sample metadata and mapping statistics for all aDNA samples described in this study. Mapping statistics include the mean depth of coverage, the mean edit distance (MQ > 30), and the percent of sequence coverage at depths of coverage one, three, and five. Further, we describe the percentage of endogenous human DNA, based on our mappings to the human genome, and expected integration sites for the recovered viral genomes based on our phylogenetic analysis (Figs. 3 and 4) and on Aswad *et al.* (*16*). An expanded version of this table is available in table S8. Calibrated C14 dates are rounded. OxCal (*75*) calibration plots are available in figs. S5 and S6, and uncalibrated C14 dates in table S4. Full mapping statistics are available in table S3.

Archaeological and anthropological data						Shotgun data			Viral mapping after target enrichment							
Sample ID	Archaeological site	Geographical location	Sample type	Rounded calibrated C14 dates	Age at Death	Genetic sex	Endogenous human DNA %	Species identified	Mean depth of coverage	Mean edit distance (MQ > =30)	% 1X	% 3X	% 5X	Deamination 5p C > T (Pos 1)	Expected HHV-6 carriage type	Expected HHV-6 integration site
utigEDI001	Edix Hill	England, Cambridgeshire	Tooth root, bone	545–640 cal CE	16–18 years	XY	46.59	HHV-6B	16.59	0.82	99.3	93.4	90.2	22%	ici	19q
utigEZH008	Ezhol	Russia, Komi Republic, Kortkerossky district	Incus bone	75–220 cal CE	ca 0.50 years	XY	75.42	HHV-6B	8.11	0.62	97.8	80.1	70.9	26%	ici	17p
utigJDS067	St Johns	England, Cambridgeshire	Tooth root	1280–1390 cal CE	9–10 years	XY	3.72	HHV-6A	3.42	1.14	84.7	32.5	20.5	22%	ici	17p
utigKRJ003	Karja cemetery	Estonia, Saaremaa	Tooth root	1175–1275 cal CE	30–35 years	XY	24.03	HHV-6A	10.33	1.11	96.5	90.3	86.7	14%	ici	18q
utigKUU029	Kukruse	Estonia, Ida-Viru	Petrous bone	1300–1400 cal CE	10.5–18 months	XX	83.33	HHV-6B	1.85	0.49	43.4	1.8	1.3	13%	NA	NA
utigOLV143	Sint-Truiden	Belgium, Limburg	Tooth root	770–975 cal CE	Adult	XX	78.04	HHV-6A	10.154	0.96	97.00	89.94	86.10	16%	ici	18q
utigOLV225	Sint-Truiden	Belgium, Limburg	Tooth root	995–1155 cal CE	20+ years	XX	83.07	HHV-6B	26.821	0.5	99.73	98.61	98.28	12%	ici/ci	NA
utigOLV303	Sint-Truiden	Belgium, Limburg	Tooth root	665–775 cal CE	20–40 years	XX	69.69	HHV-6A	6.772	1.05	95.44	78.25	67.35	15%	ici	19p
utigOLV370	Sint-Truiden	Belgium, Limburg	Tooth root	995–1155 cal CE	20–40 years	XY	23.13	HHV-6A	6.902	0.98	95.70	78.02	66.60	15%	ici	18q
utigORD001	Ordona	Italy, Apulia, Foggia	Petrous bone	780–540 cal BCE	9–11 years	XX	3.9	HHV-6B	1.77	1.59	40.1	4.6	3.2	10%	NA	NA
utigVAL004	Valjala Church Yard	Estonia, Saaremaa	Tooth root	1045–1260 cal CE	40–50 years	XX	68.27	HHV-6A	11.39	1.14	96.9	91.6	89.3	16%	ici	18q

### Host DNA analysis reveals high endogenous DNA content in most samples

The endogenous human DNA content was below 5% for JDS067 and ORD001 but above 20% for all other samples and above 65% for six samples (see table S5). The average genomic coverage was below 0.1× for three samples (ORD001, JDS067, and KRJ003), above 1× for another four samples (OLV143, OLV303, OLV225, and EDI001), and between the two for the rest. We scanned our mappings for clinical variants of interest in the context of an HHV-6 carriage but did not detect any clear occurrence of such (see Supplementary Text and table S6).

### Tissue tropism and sampling significance for HHV-6 carriage type

For this study, the tissues and anatomical sites from which the viral sequences were recovered are highly relevant as this could help evidence the type of viral carriage recovered from each host (see Table 1 and table S7). For JDS067, VAL004, KRJ003, and all OLV individuals, the only available sample types were teeth (see table S8), allowing for the possibility of both acquired and inherited infections. However, for ORD001 and KUU029 HHV-6 sequences were recovered from petrous bones and for EZH008 from an incus bone. These bones are very dense with low remodeling potential and vascularization, making an acquired active infection or a localized somatic integration or latency rather unlikely. Instead, these sampling sites point to the possibility of inherited carriage of the viruses. EDI001 was a singular case for which multiple sample types were available (tooth and bone), and the virus could be detected in all sample types, although in differential amounts, making a clear case for an inherited germ line integration in this individual.

### Comparative analysis and taxonomic classification show clear species identifications

Upon identification of HHV-6 sequences in screening shotgun datasets and enrichment (see Materials and Methods), all datasets were mapped to the HHV-6A (NC_001664.4) and HHV-6B (NC_000898.2) reference sequences (~90% sequence identity). Additional mappings to the phylogenetically closest herpesvirus, HVV-7 (NC_001716.2), were also performed to exclude potential misidentifications (see Supplementary Text), of which there were none. On the basis of the mapping statistics and coverage, samples were classified into HHV-6A and HHV-6B samples, which coincided in all cases with the original taxonomic classification using KrakenUniq (see Table 1) (*28*). We further extracted all mapped reads to respective HHV-6 reference sequences (≧MQ30) and classified them using KrakenUniq. Our results show that more than 99% of all classified reads (at the species level) were assigned to HHV-6 species for all samples (see fig. S20), demonstrating that our mappings are not affected by metagenomic contamination.

### Phylogenetic analysis yields termini ante quem for iciHHV-6 clades

**Fig. 2.**
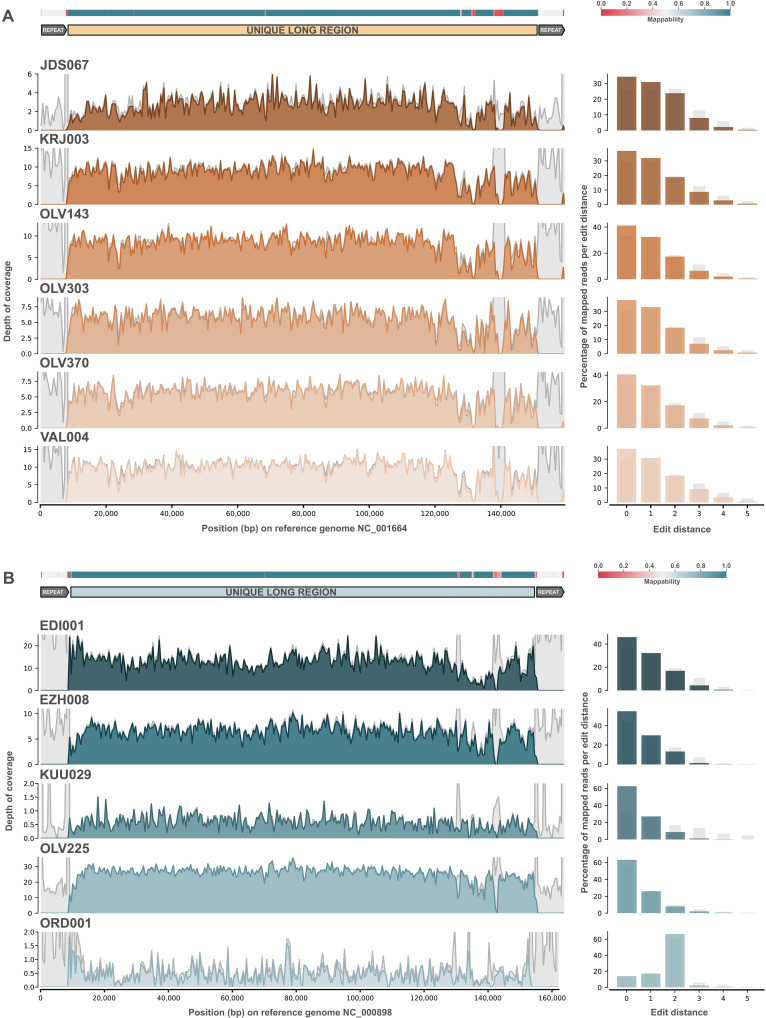
Genome coverage for all 11 ancient HHV-6 genomes reconstructed in this study. Left: Genomic coverage plots for all (**A**) HHV-6A and (**B**) HHV-6B mappings to respective reference sequences. Intervals with reads under a mapping quality of 30 are shown in light gray. On the top of each figure, mappability of the reference sequence is plotted in a heatmap and the second line shows the location of the unique long region of the 6A and 6B reference genomes used for analysis. On the right, edit distances for the mappings are shown in a barplot with light gray bars showing the percentage of reads under a mapping quality of 30.

Of the 11 ancient HHV-6 genomes recovered, 9 achieved depths of coverage high enough to generate high-quality single-nucleotide polymorphism (SNP) calls and perform a phylogenetic analysis (see table S3). The lowest coverage sample, for which we still recovered a full genome, JDS067, had a mean depth of coverage of 3.42×. All other full-genome samples were above 5× (between 6.76× and 26.56×) (see Table 1 and Fig. 2). Partial genomes, under 3×, were not used for phylogenetic analysis (KUU029 and ORD001). Following reference mapping for HHV-6A and HHV-6B, respectively, SNPs were called using freebayes (*29*) and consensus sequences were produced using bcftools (*30*) while masking for terminal repeats and repetitive/low complexity intervals (see Supplementary Text). A total of 278 modern genomes (see table S9 and the Supplementary Materials) from National Center for Biotechnology Information (NCBI) were used for the analysis. The concatenated alignments were checked in RDP5 (*31*) using the phi-test, which revealed “very good evidence for recombination,” as expected for HHV-6 viruses. Recombinant intervals were then identified and masked in the alignment using RDP5 (see Supplementary Text). Informative sites at 95% deletion were kept, resulting in an alignment length of 1408 base pairs (bp) for HHV-6A (*n* = 72) and 1942 bp for HHV-6B (*n* = 217). Maximum likelihood phylogenies were generated with IQ-Tree2 (*32*) using ultrafast bootstrap and SH-aLRT support calculations with 10,000 replicates each. IQ-Tree2’s integrated ModelFinder selected the TVM+F+ASC and TVM+F+ASC+R3 models for HHV-6A and HHV-6B, respectively.

**Fig. 3.**
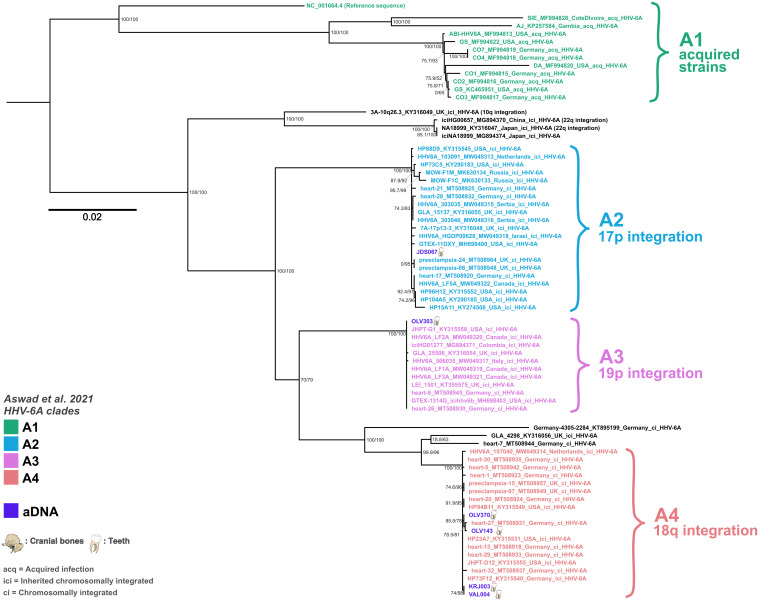
Midpoint-rooted maximum likelihood phylogeny for HHV-6A using 66 modern and 6 ancient genomes. Node labels show SH-aLRT support (%)/ultrafast bootstrap support (%) based on 10,000 replicates each in IQ-Tree2. Tip labels are colored on the basis of clade groupings, and integration locations are shown as defined in Aswad *et al.* (*16*). aDNA samples are shown in purple, with illustrations of the type of samples the genomes were extracted from. Strains are only noted as ici if they are known to be inherited.

**Fig. 4.**
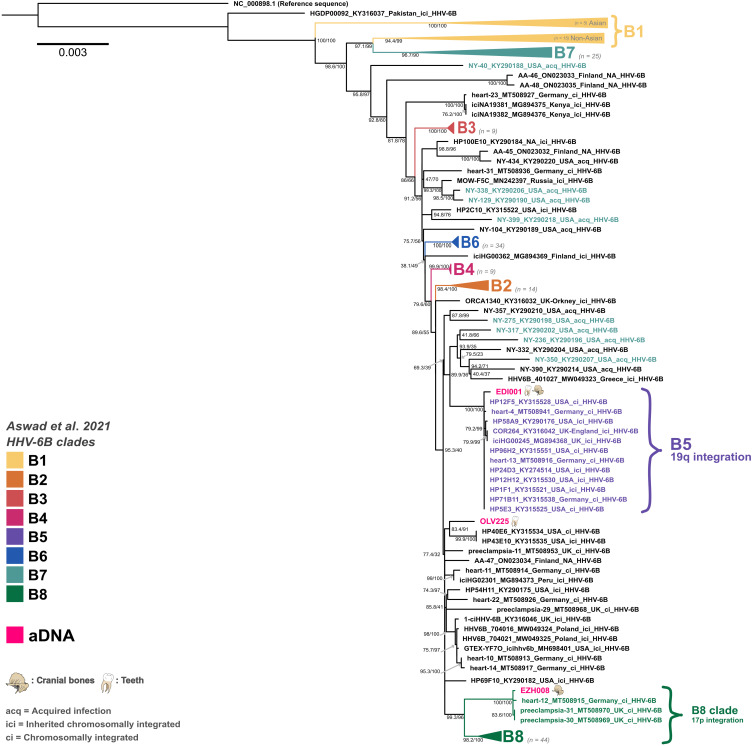
Maximum likelihood phylogeny for HHV-6B using 214 modern and 3 ancient genomes. Rooted on the reference sequence. Node labels show SH-aLRT support (%) / ultrafast bootstrap support (%) based on 10,000 replicates each in IQ-Tree2. Tip labels are colored on the basis of clade groupings, and integration locations are shown as defined in Aswad *et al.* (*16*). aDNA samples are shown in pink, with illustrations of the type of samples the genomes were extracted from. Strains are only noted as ici if they are known to be inherited.

Overall, the phylogenetic position of all ancient genomes is well supported and clearly nested within known integration clades (see Figs. 3 and 4), which show distinct evolutionary dynamics due to their replication within the human genome and therefore showcase extremely low mutation and recombination rates when compared to nonvertically inherited viruses. These clades can also give us insights into the likely integration site for each strain (*16*). An exception is sample OLV225, which is not nested in an extant defined clade associated with known human genome integration loci. This genome clusters with two ci/ici genomes from relatives from the United States with likely European ancestry (HP40E6 and HP43E10), which could hint at undersampled diversity in this lineage, as no other iciHHV-6B genomes from this clade are known today. However, this clade, situated basally on the branch giving rise to the B5 and B8 clades, is less well supported than the integrated clades and more sensitive to pruning during recombination analysis. While integration cannot be excluded for this sample because of relatively high host DNA content in the datasets, the sample type (tooth) and the strain’s phylogenetic placement do not irrevocably point toward an ici or ci strain. In contrast to Aswad *et al.* (*16*), sample NY-390 does not cluster with the B5 clade in our analysis, which seems in line with it being an acquired strain and potentially not from a host of likely European ancestries, contrary to all other B5 genomes. In our analysis, clade B1 was further split into two subgroups: one exclusively populated by Asian genomes and a second with non-Asian genomes, which is closer to the main B7 clade. Some highly divergent genomes, such as NY-310, were omitted from the main phylogeny to avoid reducing the analyzable sequence further.

On the basis of our results, we can observe that all integrated HHV-6A clades are represented in the European population by the 14th century CE at the latest but likely have a much older evolutionary history. Clade A2 is represented by one English genome dating to the 13th to 14th century CE, clade A3 by one Belgian genome dating to 7th to 8th century CE, and clade A4 is represented by four genomes from Estonia and Belgium dating between the 8th and the 13th century CE. For HHV-6B, our phylogeny yielded termini ante quem of the 1st to 6th and 6th to 7th century CE for clade B8 and B5, respectively. While we could only detect HHV-6A genomes in individuals who died after the 7th to 8th century CE, which is in contrast to the assumption that the HHV-6A species is older than the HHV-6B species, this is likely due to sampling biases rather than to unexpected emergence dates, which will likely be resolved by the addition of more ancient genomes in the future.

### Lack of global detectable temporal structure

We additionally set out to ascertain whether we could recover meaningful temporal information in the alignments through the inclusion of ancient genomic observations of HHV-6A and HHV-6B. Through consideration of topologies of mixed evolutionary behavior (concurrently considering acquired and integrated clades) and those expected to exhibit a consistent evolutionary behavior (acquired only or by integrated clade), we were consistently unable to recover a robust pattern of temporal evolution (see Supplementary Text). The exception was the HHV-6A clade of A2 integrated viruses where we were able to obtain a temporal correlation, although noting this was predominantly driven by the placement of JDS067, our lowest-coverage full genome, which was itself of uncertain topological position, either slightly basal to the A2 clade or within it (see Fig. 3 and fig. S15 to S17), depending on the analysis and length of the final alignment. Hence, we did not proceed to formal tip-calibration approaches. While not able to leverage phylogenetic information for estimation of divergence times, a major advantage of the inclusion of aDNA samples is that it allows the establishment of the absolute minimal age of a clade based on sample-associated radiocarbon information.

### Likely iciHHV-6B carrier probably succumbed to the plague

From the 11 individuals in which we could detect HHV-6 sequences, EDI001 is known to have been infected by another pathogen. A plague infection had previously been reported for this individual in Keller *et al.* (*33*). The sample stems from the site of Edix Hill, UK, which is well known for its plague cases (*33*), dating back to the first plague pandemic, and has yielded coinfections in the past (*34*). As we expect EDI001 to have carried an iciHHV-6B strain, it is unlikely to have been involved in the death of the individual, as they likely died of a *Yersinia pestis* infection that kills rapidly.

## DISCUSSION

The HHV-6 viruses are a relatively recent addition to our knowledge of herpesviruses and endogenous viruses overall. Hence, we are only beginning to piece together how and for how long they have affected human health. Some symptomatic presentations, such as the common childhood disease roseola infantum, are better understood, but the impact of integration into human genomes (somatic or germline), and particularly the transmission of integrated variants via the germ line, are still hard to elucidate. An additional by-product of their novelty in research is that no samples predating the 1980s are available, thus reducing the temporal range and signal available for evolutionary estimates even more.

### aDNA can shed light on the evolution of HHV-6A and -6B

aDNA provides the unique opportunity to bridge this gap and study DNA viruses over their history as they evolved within and with human populations [e.g., herpes simplex virus 1 (*24*), hepatitis B virus (*35*) and variola virus (*36*)]. In this study, we reconstruct nine full and two partial HHV-6 genomes from individuals dating between the 8th to 6th century BCE and the 14th century CE, expanding our knowledge to more than 2500 years of these viruses’ evolutionary histories. The genomes originate from individuals of all ages and both sexes, which were recovered from across Europe. Of these, we identify at least eight aDNA genomes to be likely iciHHV-6 genomes. For EDI001 [England, 545 to 640 calibrated (cal) CE], we were able to detect HHV-6B across multiple tissue types and the sample clustered within an endogenous clade (B5) known to integrate into Chr19q. EZH008 (Russia, 1st to 6th century CE), our second-oldest sample, clustered in an endogenous subclade (B8) known to integrate into Chr17p and was isolated from an incus bone. JDS067 (England, 1280 to 1390 cal CE), KRJ003 (Estonia, 1175 to 1275 cal CE), and VAL004 (Estonia, 1045 to 1260 cal CE) were all isolated from teeth but also clustered in endogenous clades with known HHV-6A integration loci (Chr17p for JDS067 and Chr18q for the Estonian samples).

Samples from Sint-Truiden (OLV143, OLV225, OLV303, and OLV370) stem from a large population genetics project (*37*) involving extensive sequencing (130 million raw sequence reads, on average, generated for 404 individual samples), reducing while not removing, the likelihood of large undetected groups carrying HHV-6 integrations within the population. On the basis of radiocarbon dates (see Fig. 1 and Table 1), two individuals, OLV370 and OLV225, are likely to have been contemporaneous. However, the estimation of carriage rates in aDNA datasets is complicated by two main factors: (i) the difficulties involved in obtaining and lack of large-scale contemporaneous sampling in historical populations and (ii) large variability in overall samples preservation and sequencing thresholds, leading to high amounts of false-negative samples. Nevertheless, the site confirms the concurrent presence of both HHV-6A and HHV-6B within the same population around the 10th to 12th century CE in Europe. They were all isolated from teeth. OLV143 and OLV370 cluster in the same clade as VAL004 and KRJ003 (A4). OLV303 is the only representative of the endogenous clade A3 (Chr19p), and OLV225 is the only HHV-6B sample recovered at the site and the only full genome that cannot irrevocably be placed in a known endogenous clade.

### Partial genomes can still inform us about HHV-6 endogenicity

Coverage across the human genome and the viral genome cannot be directly correlated in this study as (i) the first stems from shotgun sequencing data and the latter from enriched genomic libraries and (ii) HHV-6 viruses integrate into telomeric regions, which can complicate sequencing efforts because of variation in telomere stability/length and sequence complexity around the integration site. However, for all our samples, one pattern remained clear: Samples for which high coverage of HHV-6 genomes could be achieved and which clustered in integrated clades also showcased high levels of host DNA preservation (22.7 to 83.07%) and vice versa (see fig. S21). As we expect most of our genomes to be from germline-integrated lineages, and these results would not be expected for circulating viruses or bacteria in the context of ancient metagenomes, this pattern coincides with expected observations and persists whether genomes were isolated from petrous bones, incus bones, or teeth.

KUU029 was the exception to this pattern. The mean depth of coverage for sample KUU029 (Estonia, 1300 to 1400 cal CE) remained low, even following target enrichment (see Table 1); this sample is of interest as it might be the only acquired or somatically integrated strain in our sample set despite having been recovered from a petrous bone. Although endogenous host DNA is estimated at 83.33% for this sample, the mean depth of coverage for our mapping against the HHV-6B reference sequence is 1.88× after target enrichment. Further, the sample stems from an infant (10.5 to 18 months old), which makes the likelihood of an acquired infection higher, as today, roseola infantum is the most common active infection caused by HHV-6B (*38*) and usually manifests in children between the ages of 6 months and 2 years. The reason for the recovery of an acquired or recently somatically integrated virus from a petrous bone could be the age of the individual, as their skull would have still been in the early stages of cranial development.

### The oldest and most divergent genome: ORD001

The second sample, ORD001 (Italy, 780 to 540 cal BCE) has the lowest depth of coverage of all samples, but it is also the oldest sample in our analysis, dating to the Italian Iron Age (approximately 1100 to 600 BCE). Our mapping shows a wider divergence from the HHV-6A and HHV-6B reference sequences (mean edit distance 1.59) while still being assignable to HHV-6B based on our analysis (see Supplementary Text and fig. S13 and table S2), although its coverage remains too low for phylogenetic placement (only 40.1% of the HHV-6B reference sequence is covered at 1× before masking, following two enrichments, and no further sample is available). This is in stark contrast to the other HHV-6B samples for which the mean edit distance never exceeded 0.82. This sample likely diverged substantially from the reference sequence and showcases how dynamic and convoluted we can expect HHV-6B evolution to have been even in prehistory. This could either point to the presence of a very basal divergent form of HHV-6B or an ancestral form of HHV-6B. However, more sequencing efforts, from both modern and aDNA, are needed to determine whether an ancestral form of HHV-6B might have been circulating in the European Iron Age, as older strains and closely related modern comparative sequences (e.g., from nonhuman primates), are still missing.

### Geographical structure across the aDNA HHV-6 genomes

Geographical clustering in our phylogenies is limited within the available aDNA genomes, which could change with more data. Samples KRJ003 and VAL004 show clear geographical clustering, which is not unexpected considering that they stem from the Island of Saaremaa in the Baltic Sea (Estonia) and are of similar age (12th and 13th and 11th to 13th century CE). The same integrated clade (A4) also harbors two genomes from the Belgian site of Sint-Truiden (OLV143 and OLV370) dating to the 8th and 10th and to the 10th to 12th century CE, respectively. These groupings clearly show that clade A4 was already well dispersed in Europe and was even established in the rather remote Saaremaa population by the 11th to 13th century CE. All other high-coverage genomes (EDI001, EZH008, JDS067, OLV225, and OLV303) cluster in separate clades on their own, highlighting the heterogeneity of our sample set.

### HHV-6A no longer integrates into the genomes of populations with European ancestry

The HHV-6A phylogeny is clearly separated into circulating and endogenous genomes, with no known overlap and long branches separating each clade. One of the big open questions regarding the evolution of HHV-6A is whether the virus still integrates into the human genome or whether all integration events are ancestral (*16*, *19*), as has been hypothesized by Aswad *et al.* (*16*) on the basis of currently available modern diversity. Contrary to HHV-6B where the evolutionary line between circulating and endogenous viruses is much more blurry, host-pathogen evolution might have affected its ability to integrate into the germ line sometime in human history (*16*). On the basis of our ancient data, we can demonstrate that all known main endogenous clades (A2, A3, and A4) were already represented in historical populations. This shows that the sampled diversity found within populations of European ancestry today is in all likelihood limited to ancestral integration events and that these events are no longer occurring in European populations or only occurring in rare, undetected events. While we do not have much data for Asian strains, a small seemingly monophyletic clade of iciHHV-6A from eastern Asia is known to integrate into Chr22q and based on prior studies is also likely to stem from a single ancestral founder event, further validating this trend for the species (*19*, *39*).

Overall, the current HHV-6A phylogeny suffers from a heavy geographical sampling bias, with most of its data stemming from individuals with European ancestry, making any assumption on a global level difficult. This is further exacerbated by varying carriage rates for iciHHV-6A within populations (*19*), which are also generally lower than for iciHHV-6B [they make up only 10 to 40% of iciHHV-6 (*17*)]. However, in our case, sampled ancient and modern diversity overlap, and we can see a clear temporal continuity with ancient integration events leading to eventual long-term endogenization of the virus without reaching fixation, which were all in place at the latest between the 7th and 14th century CE. We can therefore confirm that endogenous and circulating HHV-6A genomes diverged in early human history, with clades A2 to A4 originating from ancestral founder events into the human germ line and that these integration events occurred in Europe before the colonial era.

### The evolution of HHV-6B was already convoluted in the human past

The evolutionary history of HHV-6B is much more convoluted, and prior studies have demonstrated that endogenous strains of the virus likely serve as an endemic reservoir for horizontal transmission and integration events that likely still occur to this day (*16*, *22*). This means that endogenous and circulating strains are more closely related and interspersed across the phylogeny than for HHV-6A. Our ancient HHV-6B genomes cluster in integrated clades B5 and B8. All genomes for which the donor’s ancestry is known in clade B5 are likely to be of European ancestry. Clade B8 is also predominantly populated with genomes stemming from donors of likely European ancestry, with one exception being the donor for HP46B12, for which African ancestry is assumed on the basis of previously published data (*40*). The last ancient HHV-6B strain clusters near two known endogenous genomes (HP40E6/HP43E10), which stem from first-degree relatives from North America with likely European ancestry (*16*, *41*). While it could theoretically hint at the presence of an undersampled integrated clade, this grouping is not as well supported as other integrated clades, experienced more pervasive recombination, and shows higher sequence divergence when compared to other integrated clades (which are all known to exclusively carry iciHHV-6 strains and are phylogenetically well defined). The sample is likely nested within a complex basal branch, giving rise to the clades B5 and B8 (both populated by aDNA samples), the HP40E6/HP43E10 cluster and an amalgamation of acquired and endogenous strains. It can be assumed that the clades B5 and B8 originated from ancestral integration events much like all main iciHHV-6A clades, with our earliest terminus ante quem for iciHHV-6B dated to the 1st to 6th century CE and our oldest HHV-6B sample dating to the 8th to 6th century BCE. While we were unable to formally date the age of integration of clades, even upon inclusion of ancient observations, we anticipate that it is likely that, with additional data to support a within clade sampling time series or the detection of much older observations, this may be a feasible approach to assess long-term evolutionary rates in the viral species.

### How can we study HHV-6 viruses using aDNA?

While HHV-6 viruses are ubiquitous today, they mostly remain latent somatically and are thus much harder to detect and reconstruct using hard tissue samples, which are the most common sample types for aDNA analysis. On the other hand, iciHHV-6 would theoretically be much better suited for detection in aDNA datasets, as we can expect to find the virus in every human cell from birth and high viral loads in all tissue types. However, chromosomally integrated carriage is much less prevalent, with only an estimated 0.4 to 1% of the human population being carriers today (*4*, *19*). This percentage can be estimated to grow even smaller the closer we come temporally to the endogenization of the virus into the human germ line.

On the basis of our data and the latency mechanism of the virus, we conclude that aDNA detection of HHV-6 viruses is indeed most likely to occur in the case of chromosomally integrated variants, as most of our recovered genomes cluster in endogenous clades or are likely to be integrated, despite iciHHV-6 genome carriage being much rarer than wild-type infections. This means that aDNA datasets will in all likelihood be biased toward the retrieval of iciHHV-6 but not restricted to it, as suggested by sample KUU029. The reconstruction of aDNA HHV-6 sequences is facilitated by the fact that it can be retrieved from high endogenous DNA samples, provided that host DNA is well preserved, as it is part of the human genome and replicates with it. This is also the case in sample types that are only rarely thoroughly metagenomically analyzed, which is exemplified by samples EDI001 (maxilla), KUU029/ORD001 (petrous bone), and EZH008 (incus bone). A second possibility would be the recovery of viral sequences originating from a somatic integration, e.g., into the salivary glands, from where it could have reactivated and shed into the oral cavity, as HHV-6B is known to be detectable in the saliva of healthy individuals well after their primary infection (*4*). However, HHV-6 might still often remain undetected because of bad overall DNA preservation, and their tendency to integrate into short telomeres might further affect their retrieval. Despite estimated tens of thousands of aDNA samples having been metagenomically analyzed over the past decade, this study reveals the presence of full HHV-6 sequences from archaeological populations, both demonstrating that the virus was indeed circulating in historical populations and that the virus is detectable and retrievable from aDNA datasets. The prior lack of HHV-6 data could be due to a multitude of additional factors such as: (i) reduced carriage rates in historical populations; (ii) unknown divergent lineages, which reduced detectability; (iii) low sequencing thresholds; and (iv) lack of metagenomic analysis for samples such as petrous bones or incus bones. Sequencing thresholds in the field have been increasing in recent years, but the detection of viral sequences often start with less than 10 sequencing reads being identified during metagenomic analysis, which can easily be missed at lower depths of sequencing. Thus, aDNA viral genomes remain scarce in most cases and their detection is often impeded. Nevertheless, this study demonstrates that aDNA is a powerful tool for studying HHV-6 endogenization over the entirety of human history, and it informs us on better detection strategies for HHV-6 sequences in the future.

Our data show that HHV-6A and 6B were already well established in human populations before the 14th century CE, with our oldest genome ORD001 dating to the Italian Iron Age, around the 8th to 6th century BCE, clearly showcasing the long evolutionary history of these endogenous viruses in the European populations. We provide absolute minimal ages for all defined endogenous iciHHV-6A clades and conclude that the virus is no longer integrating into the germ line in populations of European ancestry. Further, we demonstrate that HHV-6B was already present in the European Iron Age. We find that evolutionary dynamics seemed to have remained unchanged at least from the 1st to 6th century CE until today, with HHV-6B having a more convoluted evolution without a distinct line between circulating and endogenous diversity as opposed to HHV-6A, for which aDNA genomes are distinctly segregated by integration loci. Last, this study clearly demonstrates the power of aDNA research to study the evolution of HHV-6 viruses and other endogenous viral elements, which is largely unexplored in paleogenomics and opens many research questions to the field in the future. More data, particularly from early human history but also from a wider geographic range, will be key to further our understanding of the emergence and the endogenization of these viruses, which we now know have accompanied us since at least the Iron Age.

## MATERIALS AND METHODS

### Experimental design

All samples stem from skeletal human remains recovered from archaeological sites situated in Europe. Samples were not selected a priori for screening for HHV-6 species but are part of larger human population genetics projects (~4000 samples) and untargeted screening efforts to detect microbial pathogens in historical populations. Hence, most of the samples are teeth, with the exception of one incus bone, two petrous bones, and a maxilla fragment (see fig. S4). Accordingly, only genomic libraries for which we could confidently identify reads matching HHV-6 species in shotgun screening datasets from the projects mentioned above were selected for target enrichment, genome reconstruction, and further analysis, and included in this study. Samples for which no hits for HHV-6 species were identified were not included in this study.

### Laboratory work

#### Sample preparation and DNA extraction

Apical tooth roots were cut off with a drill and used for extraction since root cementum has been shown to contain more endogenous DNA than crown dentine (*42*). The root pieces were used whole to avoid heat damage during powdering with a drill and to reduce the risk of cross contamination between samples. Contaminants were removed from the surface of tooth roots by soaking in 6% bleach for 5 min, then rinsing three times with milli-Q water (Millipore), and, lastly, soaking in 70% ethanol for 2 min, shaking the tubes during each round to dislodge particles. Last, the samples were left to dry under an ultraviolet light for 2 hours. Petrous bones were sampled by drilling or cutting a sample from the core, and if inner ear bones were present, these were used instead. Petrous core and inner ear bones were decontaminated the same way as tooth roots.

Next, the samples were weighed, [20 * sample mass (mg)] μl of EDTA and [sample mass (mg)/2] μl of proteinase K was added, and the samples were left to digest for 72 hours on a rotating mixer at room temperature to compensate for the smaller surface area of the whole root compared to powder. The DNA solution was concentrated to 250 μl (Vivaspin Turbo 15, 30,000 MWCO PES, Sartorius) and purified in large volume columns (High Pure Viral Nucleic Acid Large Volume Kit, Roche) using 2.5 ml of PB buffer, 1 ml of PE buffer, and 100 μl of EB buffer [MinElute polymerase chain reaction (PCR) Purification Kit, QIAGEN].

#### Library preparation

Double-stranded libraries were built using a protocol modified from the manufacturer’s instructions of the NEBNext End Repair Module (E6050L, NEBNext), NEBNext Quick Ligation Module (E6056L, NEBNext), and Illumina-specific adaptors (*43*), following established protocols (*43*–*45*). The end repair module was implemented using 30 μl of DNA extract, 12.5 μl of water, 5 μl of buffer, and 2.5 μl of enzyme mix, incubating at 20°C for 30 min. Nine samples were processed at the Ancient DNA laboratory of the Core Facility at the Institute of Genomics, University of Tartu. JDS067 and EDI001 (tooth sample) were processed at the Ancient DNA laboratory of the Department of Archaeology, University of Cambridge and instead of 30 μl, 50 μl were input for library preparation. The samples were purified using 500 μl PB and 650 μl of PE buffer and eluted in 30 μl EB buffer (MinElute PCR Purification Kit, QIAGEN). The adaptor ligation module was implemented using 10 μl of buffer, 5 μl of T4 ligase, and 5 μl of adaptor mix (*43*), incubating at 20°C for 15 min. The samples were purified as in the previous step and eluted in 30 μl of EB buffer (MinElute PCR Purification Kit, QIAGEN). The adaptor fill-in module was implemented using 12,2 μl of water, 5 μl of buffer, 0.8 μl of deoxynucleotide triphosphate (dNTP; 25 mM each; Thermo Fisher Scientific), and 2 μl of Bst DNA polymerase, incubating at 37°C for 30 min and at 80°C for 20 min. The libraries were amplified, and combinatorial indexes (NEBNext Multiplex Oligos for Illumina, New England Biolabs) were added by PCR using HGS Diamond Taq DNA polymerase (Eurogentec). The samples were purified and eluted in 35 μl of EB buffer (MinElute PCR Purification Kit, QIAGEN). Three verification steps were implemented to make sure that library preparation was successful and to measure the concentration of dsDNA/sequencing libraries—fluorometric quantitation (Qubit, Thermo Fisher Scientific), parallel capillary electrophoresis (Fragment Analyser, Agilent Technologies) or automated electrophoresis (Agilent TapeStation system, Agilent Technologies), and quantitative PCR (KAPA Library Quantification Kit - Illumina platforms).

#### Target enrichment

Libraries, which contained HHV-6 sequences during shotgun screening, were enriched using a Daicel Arbor Biosciences Custom myBaits multispecies viral capture kit, which contained, among others, sequences for all human herpesviruses, including 193 HHV-6 sequences and four HHV-7 sequences. Nine libraries were reamplified using KAPA Hifi Hotstart ReadyMix (2×) and primers IS5 and IS6 (*43*) before enrichment (noted as SG1R input libraries in table S10). Samples EDI001, EZH008, JDS067, and ORD001 were enriched using the v4 myBaits reagent kit and samples VAL004, KRJ003, KUU029, OLV143, OLV225, OLV303, and OLV370 with the v5 myBaits reagent kit. Samples were each enriched in a single reaction with 2.75 μl of baits per reaction using relatively low hybridization temperatures (61° to 62°C) to allow for divergence from the template. Enriched libraries were amplified using KAPA HiFi HotStart ReadyMix (2×) DNA polymerase and primers IS5 and IS6 (*43*). For samples ORD001 and JDS067, previously enriched libraries were used for a second round of capture (see table S10) due to low initial yield. Following amplification, the libraries were sequenced on an Illumina NextSeq500 sequencing platform (MID150, PE) at the University of Tartu, Institute of Genomics Core Facility, with other capture samples.

### Statistical analysis

#### Human betaherpesvirus 6 genomic analysis

##### Ancient datasets: Raw FASTQ preparation

Raw sequencing data (see table S10) were merged by library and type of data (shotgun/capture). Data quality was assessed with fastqc (*46*). For paired-end data, cutadapt (*47*) was run in paired-end mode with the pair-filter on “any,” quality trimming was set to 20, and reads under 20 bp were discarded (--times 3 -e 0.2 -j 0 --trim-n) [for the metagenomic screening, only reads above 30 bp were kept for both paired-end (PE) and single-end (SE)]. Reads were merged using Flash2.0 (*48*). Single-ended datasets were also trimmed and quality filtered using cutadapt (-m 20 --nextseq-trim= 20 --times 3 -e 0.2 -j 0 --trim-n). Dataset quality was assessed with multiqc (*49*).

##### Ancient datasets: Metagenomic screening

Following data filtering, trimming and, if needed, paired-end read merging, samples were analyzed using the taxonomic classifier KrakenUniq (*28*) to detect the presence of human-associated pathogens. The database used for the analysis was composed of the following: dusted complete genomes and chromosome-level assemblies of bacteria, viruses, archaea, and protozoa; the human genome; the NCBI Viral Neighbor database; and the contaminant databases UniVec and EmVec. Heatmaps were computed using plotly (*50*), pandas (*51*), matplotlib (*52*), and numpy (*53*). *E* values were calculated as follows: Kmer CountRead Count×Coverage, where “Coverage” is the coverage across the taxon kmer dictionary. The *E*-value cutoff to choose taxa for further inspection was 0.001. Taxa identifications were then verified by mapping the data to appropriate reference sequences as detailed for our final alignments.

##### Modern datasets: Data preparation and screening

Modern datasets were downloaded from NCBI and fragmented using the script FASTA-FRAG (*54*) into 50-bp fragments tiled across the sequences 20× with two base pair gaps. Fragments were analyzed with kraken2 (*55*) to verify the species associated with the samples.

##### Comparative mappings

Following the identification of the pathogens, target enrichment, and further sequencing, datasets were merged by library and separately mapped to the HHV6-A (NC_001664.4), HHV-6B (NC_000898.1), and HHV-7 (NC_001716.2) reference genomes (see figs. S10 to S13) using bwa aln (-n 0.04 -l 1000) (*56*) with samse for single-end data and merged paired-end reads. SAM files were converted to BAM format, sorted, indexed, and filtered for mapped reads using samtools (*57*). Picard (*58*) was used to remove duplicates with the MarkDuplicates module. Following duplicate removal, datasets were merged by sample. Misincorporation patterns were computed, and recalibration was done using mapDamage (v2.2.1) (*59*). Mapping plots were visualized using aDNA-BAMPlotter (*60*), and mapping statistics were computed using Qualimap (*61*) and pysam (*62*), pandas (*51*), biopython (*63*), and numpy (*53*).

##### Phylogenetic analysis

Following mapping, consensus sequences were generated using freebayes (v.1.3.5) (*29*) and bcftools (v1.16 using htslib v1.6) (*64*) with either the reference sequence for HHV-6A or HHV-6B based on the species identification for each sample. Using deduplicated and rescaled BAM files for aDNA samples, SNPs were called with the following options: --max-complex-gap -1 --report-monomorphic -q 30 --min-alternate-count 3 --min-coverage 3 -m 30 -F 0.9 --ploidy 1. For modern samples, deduplicated BAM files were used with the following options: --max-complex-gap -1 --report-monomorphic --min-alternate-count 5 --min-coverage 5 -m 30 -F 0.9 --ploidy 1. VCF files were then filtered with bcftools filter with the options “-s LOWQUAL -i '(QUAL>= 30 && INFO/DP>= 3) |(FORMAT/QR>100 && INFO/DP>=3)'” for aDNA samples and “-s LOWQUAL -i '(INFO/DP>=10)'” for modern DNA samples. Indels were removed. Filtered VCF files were then compressed using bcftools view and indexed using bcftools index.

Using bedtools intersect (*65*), SNPs which were only reported for aDNA samples were identified and visually inspected using IGV (*66*). This was also done for C > T and G > A variants to exclude possible aDNA misincorporations to be included in the final SNP alignment. Consensus sequences were called using bcftools consensus “-a “N” --exclude 'FILTER= “LOWQUAL”'” for modern samples and “-a “N” --exclude 'FILTER= “LOWQUAL” | INFO/DP<3'” for aDNA samples. Masking was applied for both modern and aDNA sequences to exclude repetitive intervals (see Supplementary Text). Initial masking for the reference sequences for HHV-6A (NC_001664.4) and HHV-6B (NC_000898.1) was done on the basis of the annotation provided by NCBI, by masking repeats. In addition, these intervals were corrected by running GenMap (*67*) on both reference sequences to adjust the interval coordinates. Intervals with low mappability preceding and following known repetitive regions were also included in the mask (see Supplementary Text).

Masked consensus sequences and the reference sequence for each species were concatenated on the basis of the identified taxon. Recombination analysis was performed in RDP5 (v4.45) (*31*). Initially, alignments were checked with a quick Phi test, which revealed “very good evidence for recombination” in both alignments. Recombination intervals were identified (see Supplementary Text) and masked in the alignment. Using Mega11 (*68*), the alignment was filtered for variant positions present in 95% of all sequences. This alignment was then used to generate a maximum likelihood phylogeny in IQ-Tree2 (*32*) using the integrated “Model Finder Plus” without restrictions to choose the best-fitting models. Branch support was calculated with 10.000 ultrafast bootstrap approximation and SH-like approximate likelihood ratio test replicates.

##### Consensus building for temporal analysis

For our temporal analysis, we mapped each ancient genome to a representative genome of the clade it clustered in on the basis of our maximum likelihood phylogeny (Figs. 3 and 4). All strains, with the exception of OLV225, clustered in an integrated clade, which evolved in a clonal manner, hence with an expectation of no larger structural changes. Consistently, recombination analysis of only integrated clades also revealed no evidence for recombination (see Supplementary Text). Using the same approach as described above, consensus sequences were generated on the basis of the following reference sequences: MT508932 (clade A2), MT508930 (clade A3), MT508933 (clade A4), MT508941 (clade B5), and MT508915 (clade B8). OLV225 was mapped to its phylogenetically closest genome on the basis of our phylogeny, KY315534 (HP40E6). Mapping statistics can be found in table S11, and plots showing sequence coverage, edit distance, and deamination can be found in fig. S18. In brief, for each dataset, we conducted a core SNP calling approach followed by recombination pruning and reconstruction of maximum likelihood phylogenies. Temporal signal over these phylogenies was evaluated using linear regression implemented in BactDating (*69*), which assessed the correlation coefficient and significance following 10,000 permutations of the sampling dates. For a detailed description of the temporal analysis, please see Supplementary Text.

### Human data genomic analysis

#### Mapping

Before mapping, the sequences of adaptors and indexes and poly-G tales occurring due to the specifics of the NextSeq 500 technology were cut from the ends of DNA sequences using cutadapt 2.1 (*47*). Sequences shorter than 30 bp were also removed with the same program to avoid random mapping of sequences from other species. For paired-end sequencing data, R1 and R2 reads were merged using flash 1.2.11 (*48*). The sequences were mapped to reference sequence GRCh37 (hs37d5) using Burrows-Wheeler Aligner (BWA 0.7.17) (*56*) and command aln with reseeding disabled. After mapping, the sequences were converted to BAM format, and only sequences that mapped to the human genome were kept with samtools 1.9 (*57*). Next, data from different flow cell lanes was merged, and duplicates were removed with picard 2.20.8 (*58*). Indels were realigned with GATK 3.5 (*70*), and lastly, reads with mapping quality under 10 were filtered out with samtools 1.3 (*57*).

#### aDNA authentication

As a result of degradation over time, aDNA can be distinguished from modern DNA by certain characteristics: short fragments and a high frequency of C= > T substitutions at the 5′ ends of sequences due to cytosine deamination. The program mapDamage2.0 (*59*) was used to estimate the frequency of 5′ C=>T transitions. Mitochondrial DNA (mtDNA) contamination was estimated using the method from Fu *et al.* (*71*). This included calling an mtDNA consensus sequence based on reads with mapping quality at least 30 and positions with at least five times coverage, aligning the consensus with 311 other human mtDNA sequences from Fu *et al.* (*71*), mapping the original mtDNA reads to the consensus sequence and running contamMix 1.0-10 (*71*) with the reads mapping to the consensus and the 312 aligned mtDNA sequences while trimming seven bases from the ends of reads with the option trimBases. For the male individuals, contamination was also estimated on the basis of chromosome X using the two contamination estimation methods first described in Rasmussen *et al.* (*72*) and incorporated in the ANGSD software (*73*) in the script contamination.R.

#### General statistics and genetic sex

Samtools 1.3 (*57*) option stats was used to determine the number of final reads, average read length, average coverage, etc. Genetic sex was calculated using the script sexing.py from Skoglund *et al.* (*74*), estimating the fraction of reads mapping to Y chromosome out of all reads mapping to either X or Y chromosome.

### Provenance

The sampled teeth and bones were collected from archaeological human remains by several local archaeologists (all coauthors of this study) from archaeological sites located throughout Europe; see Supplementary Text and table S14 for details. The samples were radiocarbon dated by multiple laboratories, both for this study and from previously published work; see table S4 for details. Sampled individuals are housed at a variety of museums and institutions. Please see table S14 regarding details about their location, the people responsible for sampling, and contacts for access to the osteological material.

## Supplementary Materials

### The PDF file includes:

Supplementary Text Figs. S1 to S21 Legends for tables S1 to S14 References
